# Tumor histology is an independent prognostic factor in locally advanced cervical carcinoma: A retrospective study

**DOI:** 10.1186/s12885-022-09506-3

**Published:** 2022-04-13

**Authors:** Lenny Gallardo-Alvarado, David Cantú-de León, Rebeca Ramirez-Morales, Gabriel Santiago-Concha, Salim Barquet-Muñoz, Rosa Salcedo-Hernandez, Cinthya Reyes, Sandra Perez-Alvarez, Delia Perez-Montiel, Carlos Perez-Plasencia, Elizabeth Trejo-Duran, Juan Pablo Galicia

**Affiliations:** 1 Programa de Maestría Y Doctorado en Ciencias Médicas, Odontológicas Y de La Salud. UNAM. Mexico City, Mexico City, Mexico; 2 Subdirección de Investigación Clínica, Instituto Nacional de Cancerología, Mexico City, Mexico; 3 Dirección de Investigación, Instituto Nacional de Cancerología, Mexico City, Mexico; 4 Departamento de Radioterapia, Instituto Nacional de Cancerología, Mexico City, Mexico; 5 Departamento de Ginecología, Instituto Nacional de Cancerología, Mexico City, Mexico; 6 Departamento de Patología, Instituto Nacional de Cancerología, Mexico City, Mexico; 7 Laboratorio de Genómica, Instituto Nacional de Cancerología, Mexico City, Mexico

**Keywords:** Histology, Locally advanced, Cervical carcinoma, Prognosis

## Abstract

**Background:**

Even with different histologic origins, squamous cell carcinoma (SCC) and adenocarcinoma (AC) are considered a single entity, and the first-line treatment is the same. Locally advanced disease at the diagnosis of cervical cancer is the most important prognostic factor, the recurrence rate is high, making it necessary to evaluate prognostic factors other than clinical or radiological staging; histology could be one of them but continues to be controversial. The aim of this study was to evaluate tumor histology as a prognostic factor in terms of treatment outcomes, disease-free survival (DFS) and overall survival (OS) in a retrospective cohort of patients with Locally Advanced Cervical Carcinoma (LACC).

**Methods:**

The records of 1291patients with LACC were reviewed, all of them were treated with 45–50 Gy of external beam radiotherapy with concurrent chemotherapy and brachytherapy. A descriptive and comparative analysis was conducted. Treatment response was analyzed by the chi-square test; DFS and OS were calculated for each histology with the Kaplan–Meier method and compared with the log-rank test; and the Cox model was applied for the multivariate analysis.

**Results:**

We included 1291 patients with LACC treated from 2005 to 2014, of which 1154 (89·4%) had SCC and 137 (10·6%) had AC. Complete response to treatment was achieved in 933 (80·8%) patients with SCC and 113 (82·5%) patients with AC. Recurrence of the disease was reported in 29·9% of SCC patients and 31·9% of AC patients. Five-year DFS was 70% for SCC and 62·2% for AC. The five-year OS rates were 74·3% and 60% for SCC and AC, respectively. The mean DFS was 48·8 months for SCC vs 46·10 for AC (*p* = 0·043), the mean OS was 50·8 for SCC and 47·0 for AC (*p* = 0·002).

**Conclusion:**

Our findings support the hypothesis that SCC and AC are different clinical entities.

**Trial Registration:**

NCT04537273.

## Background

It is estimated that in 2018, over 311,000 women died from cervical cancer (CC) around the globe, with up to 90% of deaths reported in low- and middle-income countries and in minority populations in high-income countries. Most patients in these populations are diagnosed in advanced stages of the disease, and clinical stage is the most significant prognostic factor in this neoplasm [1–4].

Locally advanced cervical cancer (LACC) is a tumor whose size exceeds what can be treated successfully with surgery and includes International Federation of Obstetrics and Gynecology (FIGO) stages IB2-IVA; the primary treatment for these patients is concurrent chemoradiotherapy and has category 1 of evidence and consensus; overall survival (OS) with this treatment ranges from 56–75%, depending on the series and populations [4–12].

Two major histologic types have been described: squamous cell carcinoma (SCC) is the most common histology, representing approximately 70–75% of cases, while 10–25% of cases are adenocarcinomas (AC), which have increased in incidence in recent decades [13–16]. Even with different histologic origins, SCC and AC share many risk factors, such as HPV infection, an increased number of sexual partners, and prolonged use of oral contraceptives. In general, first-line treatment is the same for both histologies, and they are considered a single entity [17–19].

Locally advanced disease continues to be a public health problem in emergent economies. Even though treatment is very well standardized, the recurrence rate is still high, making it necessary to evaluate prognostic factors other than clinical or radiological staging, and histology could be one of them but continues to be controversial [20–33]. Therefore, the aim of this study was to evaluate tumor histology as a prognostic factor in terms of treatment outcomes, disease-free survival (DFS) and OS in a retrospective cohort of patients with LACC treated with standard chemoradiotherapy in a reference hospital in Mexico.

## Methods

This was a retrospective study, and after Institutional Review Board (IRB) approval, the data were obtained from the clinical files of CC patients with clinical stages IB2-IVA (FIGO 2009) treated at the Instituto Nacional de Cancerología in Mexico City from January 2005 to December 2014.

Two gynecologist oncologists performed information verification to ensure data accuracy in the medical records. Then the other four medical doctors compiled the data with double-check review to ensure accuracy.

A total of 1954 patients with LACC confirmed by pathology, clinical exams and computed tomography scan (CT) were identified. The exclusion criteria were adenosquamous cell carcinomas or rare histologies, such as gastric type adenocarcinoma, neuroendocrine or clear-cell carcinoma, incomplete treatment or not treated with chemoradiotherapy, two primary malignancies, or insufficient data for analysis.

We identified 1291 patients suitable for analysis, all of whom were treated with 45–50 Gy of external beam radiotherapy (EBRT) with at least three doses of concurrent platinum-based chemotherapy or gemcitabine (in case of renal dysfunction) and high or low dose rate brachytherapy (depending on the availability at the moment of treatment).

Demographic, clinical, pathological and follow-up data and the survival status of all patients were recorded. Treatment outcome was classified as complete response if the patient had no signs of tumor activity after 6 months of finishing treatment; persistence of disease was defined if tumor could be identified after treatment or before six months of treatment termination; and progression was defined if tumor growth occurred or metastatic disease appeared. DFS was defined as the period between treatment completion and relapse, which was confirmed by pathological study and/or CT. OS was defined as the period between diagnosis and death or the last visit.

Quantitative variables were described with central tendency and dispersion measures and analyzed with Student’s t test or the Mann–Whitney U test. Normality was determined with Shapiro–Wilk’s test, chi-square for categorical comparisons between groups, and Kaplan–Meier with the log-rank test for survival analysis. The multivariate analysis was performed using the Cox proportional hazard regression model. Statistically significant differences were defined as a *p* value < 0·05.

Statistical analyses were performed using SPSS, version 23 (IBM Corp., Armonk, NY, USA). The study was performed according to the Declaration of Helsinki (6th version, Seoul, South Korea, 2008) and authorized by the Comité de Ética en Investigación del Instituto Nacional de Cancerología (Rev/050/18). This Study has been granted an exemption from requiring informed consent because of the nature of the Study by the Comité de Ética en Investigación del Instituto Nacional de Cancerología (Rev/050/18).

## Results

Of the 1291 patients with LACC and complete standard treatment, 1154 (89·4%) had SCC and 137 (10·6%) had AC. The median age was 51 years for SCC (range 19–87) and 47 years for AC (range 26–78), a difference of five years (*p* = 0·023). There were no differences regarding body mass index (BMI) and performance status among groups.

In the analysis of clinical and radiological characteristics, 2 patients did not have information about tumor size, and 64 (5%) patients did not have a basal CT scan available for evaluation. AC presented, in general, in earlier stages than SCC (Table 1) (*p* < 0·0001), and parametrial involvement was more frequent in SCC (*n* = 1002; 86·8%) vs AC (*n* = 110, 80·3%) (*p* < 0·0001). We did not find differences between tumor size and pelvic lymph node status among groups (*p* = nonsignificant [NS]).

**Table 1:** Demographic and Clinicopathological characteristics (*n* = 1291)

	Squamous-Cell Carcinoma *n*(%)	Adenocarcinoma *n*(%)	*p-*value
	*n* = 1154 (89.4)	*n* = 137 (10.6)	
**Age, median (range)**	51 (19–87)	47 (26–78)	0.023
**BMI median (range)**	27 (16.1–56.7)	26.9 (17.5–41.7)	0.73
**Performance status**^**a**^			0.11
< 80%	166 (14.4)	11 (8.0)	
90–100%	983 (85.2)	125 (91.2)	
Unknown	5 (0.4)	1 (0.7)	
**FIGO Clinical Stage**			< 0.001
IB2	83 (7.2)	24 (17.5)	
IIA	63 (5.5)	3 (2.2)	
IIB	650 (56.3)	94 (68.6)	
III	332 (28.8)	14 (10.2)	
IVA	26 (2.3)	2 (1.5)	
**Tumor size, cm**			0.36
< 4 cm	348 (30.2)	49 (35.8)	
> 4 cm	804 (69.7)	88 (64.2)	
Unknown	2 (0.2)	0	
**Parametrial involvement**			< 0.001
Negative	152 (13.2)	27 (19.7)	
Positive but not up to the pelvic wall	711 (61.6)	96 (70.1)	
Extension to the pelvic wall	291 (25.2)	14 (10.2)	
**Pelvic Lymph-Node Status**			0.30
Positive	402 (34.8)	43 (31.4)	
Negative	692 (60.0)	90 (65.7)	
Unknown	60 (5.2)	4 (2.9)	
**Tumor Grade**			< 0.001
1 (well differentiated)	9 (0.8)	32 (23.4)	
2 (moderately differentiated)	861 (74.6)	87 (63.5)	
3 (poorly/undifferentiated)	284 (24.6)	18 (13.1)	
**LVSI **^**b**^			0.002
Yes	130 (11.5)	4 (2.9)	
No	1024 (88.7)	133 (97.1)	
**Treatment outcome**			0.87
Complete response	933 (80.8)	113 (82.5)	
Partial, progression or stable disease	199 (17.2)	22 (16.1)	
Unknown	22 (1.9)	2 (1.5)	
**Total Recurrence during follow-up**	*n* = 933	*n* = 113	0.61
	279 (29.9)	36 (31.9)	

^a^ Karnofsky status ^b^ lympho-vascular space invasion

In the comparison of the pathologic characteristics, we evaluated tumor grade and lymphovascular space invasion (LVSI) and found significant differences in both variables (*p* < 0·0001 for tumor grade and *p* = 0·002 for LVSI) when comparing SCC and AC.

Complete response to treatment (by clinical and CT study) was achieved in 1046 patients (81%): 933 (80·8%) with SCC and 113 (82·5%) with AC. Recurrence of the disease was reported in 29·9% of SCC patients and 31·9% of AC patients, with no differences between the groups. Demographic and clinicopathological characteristics are described in Table 1.

**Fig. 1:**
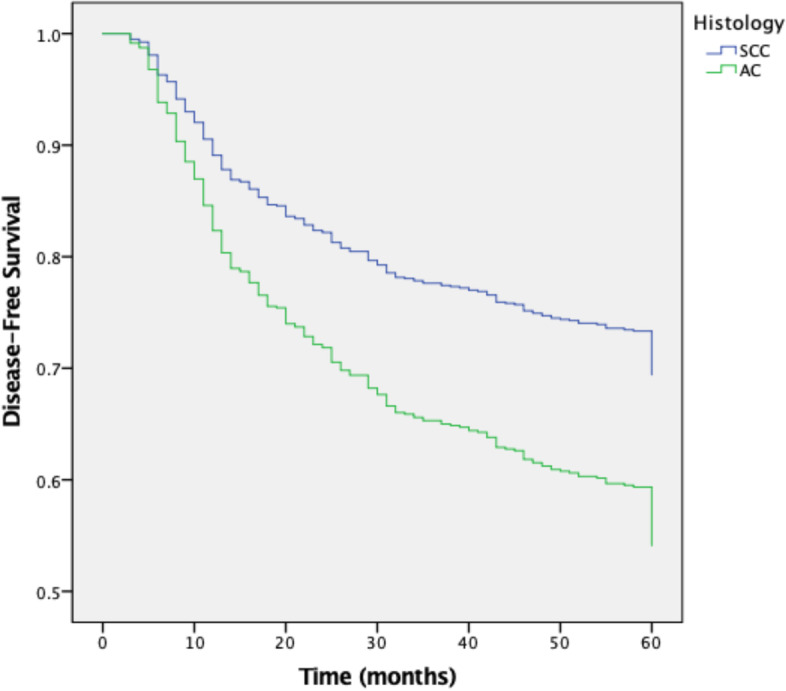
Five years’ disease-free survival curve

**Fig. 2:**
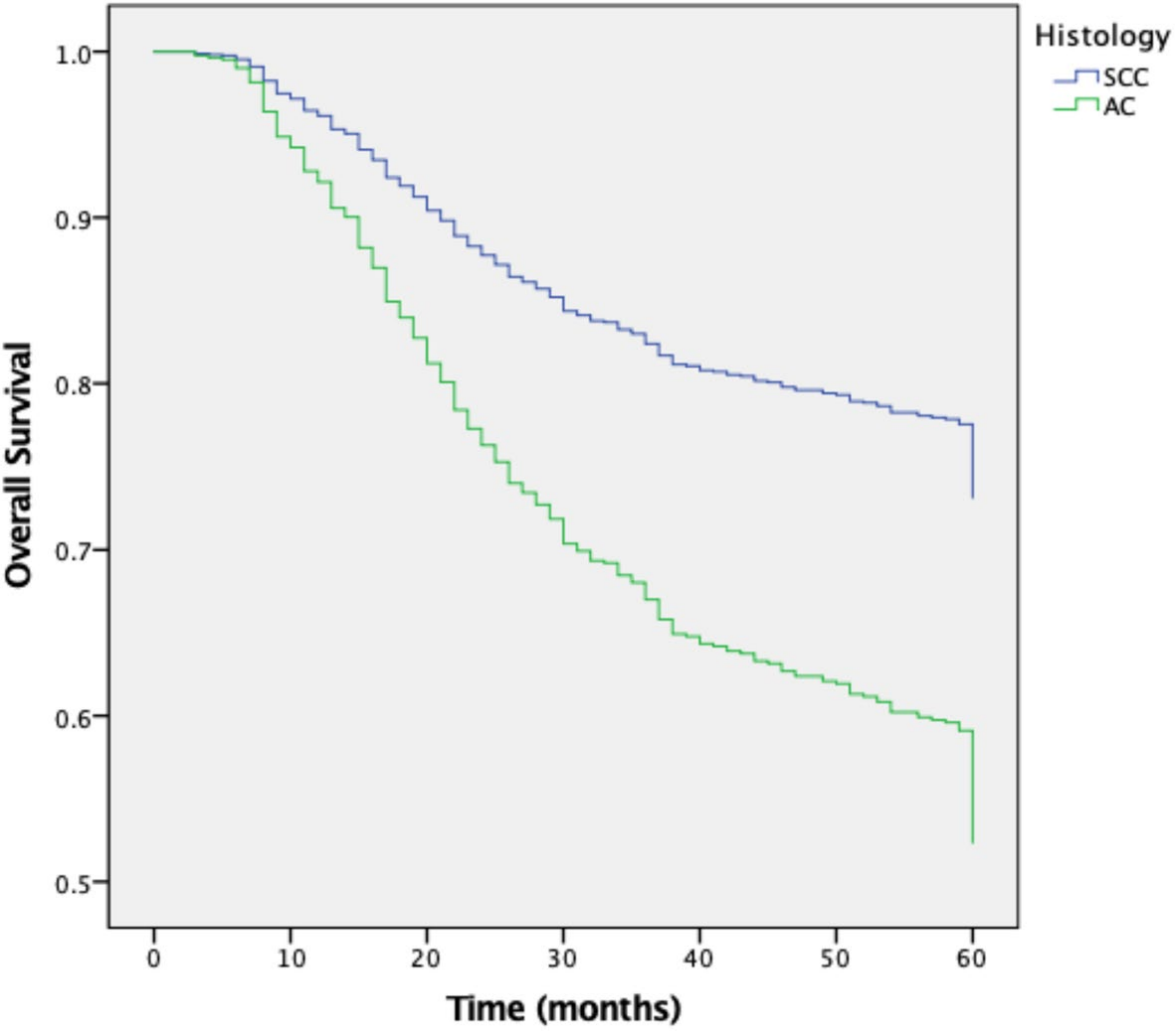
Five years’ overall survival curve

The median follow-up was 61 months (range 0–171) for SCC and 62 months (range 0–181) for AC (*p* = 0·33); the five-year DFS rates were 70% and 62·2%, respectively. The five-year OS was 74·3% and 60% in SCC and AC, respectively. The mean DFS was 48·8 months for SCC vs 46·10 for AC (*p* = 0·043), and the mean OS was 50·8 for SCC and 47·0 for AC (*p* = 0·002; Table 2 and Figs. 1 and 2).

**Table 2:** Disease-Free Survival (*N* = 1046) and Overall-Survival of Patients (*N* = 1291)

	Squamous-Cell Carcinoma	Adenocarcinoma		*p-*value
**Mean DFS**^**a**^**, months**	48.38 (47.08–49.68)		46.10 (41.68–50.21)	0.043
**Mean OS**^**b**^**, months**	50.85 (49.8–51.8)		47.07 (43.42–50.71)	0.002
**5-year DFS**	70.0%		62.2%	
**5-year OS**	74.3%		60.0%	

^a^Disease-free survival; ^b^ Overall-survival

The multivariate analysis showed that histology, tumor grade, LVSI and clinical stage were independent prognostic factors for DFS and that age, clinical stage, tumor grade, LVSI, parametrial involvement and histology were independent prognostic factors for OS (Table 3).

**Table 3:** multivariate analysis for disease free survival and overall survival (Cox Model)

	DFS^**a**^			OS^**b**^		
	**HR**^c^	**95%CI**	***p-*value**	**HR**	**95%CI**	***p-*value**
**Age**	NS		0.709	0.987	0.978–996	0.005
**FIGO Clinical Stage**	1.264	1.157–1.381	< 0.001	1.391	1.276–1.516	< 0.001
**Tumor Grade**	1.419	1.120–1.797	0.004	1.496	1.194–1.874	< 0.001
**LVSI**	0.612	0.441–849	0.003	0.598	0.439–0.814	0.001
**Parametrial involvement**	NS^d^		0.868	2.071	1.716–2.499	< 0.001
**Histology**	1.460	1.012–2.106	0.043	1.723	1.228–2.416	0.002

^a^Disease-free survival; ^b^Overall-survival. ^c^ hazard ratio; ^d^not significant

## Discussion

In this study, we found that histological type was an independent prognostic variable in patients with LACC who were treated with concomitant chemoradiotherapy. Patients with AC had a worse prognosis than those with SCC (for DFS: HR = 1·46, 95% CI = 1·012–2·106; for OS HR = 1·723, 95% CI = 1·22–2·41). There is a considerable discrepancy in the literature regarding the prognostic value of histological types. On the one hand, some reports analyzed small patient cohorts and several variables from one or a few centers; on the other hand, some reports analyzed large data sets mainly from epidemiological records that included many patients, but they did not consider all the variables that could alter the results. Most studies on this variable in LACC conclude that histological type is an independent prognostic factor (Table 4).

**Table 4:** Comparison of histology impact between studies in locally advanced cervical cancer in the last decade

**Authors**	Years of patient inclusion	Type of study	Clinical stage	*N*	SSC/AC proportion %	OS	*P*-value
**Galic et al. (2012)**	1988–2005	Retrospective multicenter	IIB-IVA	10,217	84.2/10.8/5.0^a^	32.5/17.9/29.2	0.014^b^
**Katanyoo et al. (2012)**	1995–2008	Retrospective	IIB-IVA	423	66.7/33.3	61.7/59.9	0.191
**Intaraphet et al. (2013)**	1995–2011	Retrospective	I-IV	1978	82.5/17.5	60/54.7	< 0.001
**Rose et al. 2014**		Retrospective 5 clinical trials	IB2-IVA	1671	89.1/10.9^c^	–––––––	0.459
**Chen et al. (2014)**	1995–2009	Retrospective	IIB-IVA	229	84.7/15.3^c^	58.1/41.3	0.090
**Yun Lee et al. (2015)**	1993–2012	Retrospective		3156	85.0/15.0	–––––––-	0.003
**Zhou et al. (2017)**	1988–2013	Retrospective multicenter	I-IV	8751	86/10.6/3.4^a^	51.1/40.3	< 0.001
**Yokoi et al. (2017)**	1993–2014	Retrospective	IIB-IVA	249	90.4/9.6	58.6/26.7	0.004
**Seamon et a (2017)**	1999–2012	Retrospective 3 clinical trials treated whit chemotherapy doublets	IVB^ξ^	781	77/23	10.3/15.1	0.093
**Cheng Yin et al. (2018)**	2004–2016	Retrospective Single center	IB2-IVA	181	83.4/16.5	78.6/46.0	< 0.001
**Hu et al. (2018)**	2011–2014	Retrospective	IB-IVA	815	91.2/8.7	85.2/74.4	0.005
**Jonska-Gmyrek et al. (2019)**	2004–2012	Retrospective	IIB IIIA-IIIB	161	67.7/32.3	81.7/62.8 73.1/33.6	0.03 0.005
**Current Study**	2005–2014	Retrospective Single center	IB2-IVA	1291	1154/137	74.3/60.0	0.004

^a^ Adenosquamous carcinoma ^b^calculated only for IIIB clinical Stage ^c^AC + adenosquamous carcinoma

Galic et al. performed a multicenter retrospective study that included patients with stage IIB-IVA disease who were treated between 1988 and 2005. They concluded that women with locally advanced adenocarcinoma were 21% more likely to die than those with SCC (HR = 1·21, CI 95% = 1·10–1·32) [26]. Intaraphet et al., Yun Lee et al., Yokoio et al., Cheng Yin et al., Hu et al., and Jonska-Gmyrek et al. have also described statistically significant differences in the prognosis of AC and SCC [30, 33–37]. Zhou et al. published one of the papers with the largest number of patients analyzed in the last decade. Using the SEER database, they assessed 8,751 patients and determined that AC had a worse prognosis than SCC [28]. However, Katanyoo et al., Rose et al., Chen et al., and Seamon et al. did not find differences [25, 31, 38, 39]. It is worth noting Rose and Seamon compared the differences between histological types in prospectively recruited patients in controlled trials, which improved data quality. However, their main goal was not to find differences between histological types but to evaluate the safety and efficacy of a certain treatment. Rose et al. examined 1,671 patients with LACC from five different trials, whereas Seamon et al. evaluated 781 patients with recurrent or metastatic disease from three randomized trials conducted between 1999 and 2012. Neither of these studies found differences between histological types (*p* = 0·45 and 0·093, respectively).

Tumor grade is another relevant finding that is consistent with the literature. Our study shows that more than 23% of cases of adenocarcinoma are well differentiated, in contrast to < 1% of SCC. Since this grade has a good prognosis and is more common in adenocarcinoma, it is essential to consider it in the multivariate analysis [20, 22, 26, 28, 31].

In addition to tumor grade, we found that age, clinical stage, LVSI, and parametrial involvement were independent prognostic factors in LACC. These findings have also been described in other series [22, 34].

We did not find differences among other variables, such as functional status, BMI, metastasis in the pelvic nodes, and tumor size, which are commonly described as risk factors [38, 40]. Regarding tumor size, it is likely that in locally advanced stages when tumors invade neighboring structures, the size of the initial lesion loses its prognostic value.

The limitations of our study are its retrospective design and the limited information obtained from older CT scans (2005–2008). The strength of our study is the number of patients from a single center, which partly ensures the homogeneity of treatment and staging criteria.

Other limitations of our study is the lack of HPVA tests in adenocarcinomas because the cases analyzed were diagnosed between 2005 to 2014 when the classification of adenocarcinomas was based basically on histological features, using the WHO Classification in that time. When experienced pathologists evaluate these lesions, a good correlation exists with the neoplasms associated to HPV infection, such as villoglandular and micropapillary architectural variants and the mucinous types. The tumors not associated to HPV infection are not common and immunohistochemistry or other diagnostic tools needed to confirm diagnosis are expensive, as suggested by Stolnicu S, et al., [41] and in low- and middle-income countries, where cervical cancer is more frequent, these resources are limited. Probably we need other forms to improve the classification of adenocarcinomas with a test available in the countries where cervical cancer is most prevalent [41, 42]. Now, no different strategies for treatment have been implemented in relation to the association to HPV, even though, prognosis could be worse, in the future personalized treatment based on HPV for this specific histology of cervical neoplasm might be necessary.

Although AC and SCC are distinct entities at the histological and molecular levels, several factors could account for the literature discrepancy about the role of these as a prognostic factor. Mainly published reports have been retrospective so far. They exhibit the typical bias of such study design, especially the accuracy of the collected data, availability of information including all cofounding variables that could modify the results, such as total dose of radiation therapy or number of chemotherapy cycles. In some cases, the limitations include lack of imaging tests with the inability to detect lymph node disease, and the physician expertise to perform physical examinations and classify the disease.

CC incidence has decreased over the last 40 years. Incidence rates by stage at the time of diagnosis decreased from 2001 to 2015 for SCC, but those of AC remained stable or even increased [18, 43]. Considering that the rates of AC could still increase, it is essential to determine whether the histological type is truly an independent prognostic factor requiring a special approach to improve the prognosis in patients with AC in LACC.

## Conclusion

Our findings support the hypothesis that SCC and AC are different clinical entities. Prospective studies are warranted to include histological types when developing treatments for patients with LACC. Considering the poor survival rates of patients with AC, more efficient research protocols are needed to manage this group of patients.

## Data Availability

The datasets used and/or analysed during the current study are available from the corresponding author on reasonable request.

## Abbreviations

AC: Adenocarcinoma
BMI: Body mass index
CC: Cervical cancer
CT: Computed tomography scan
EBRT: External beam radiotherapy
IRB: Institutional Review Board
FIGO: International Federation of Obstetrics and Gynecology
DFS: Disease-free survival
NS: Nonsignificant
OS: Overall survival
SCC: Squamous cell carcinoma
LACC: Locally Advanced Cervical Carcinoma
LVSI: Lymphovascular space invasion
