# Correction: Tumor histology is an independent prognostic factor in locally advanced cervical carcinoma: A retrospective study

**DOI:** 10.1186/s12885-022-09545-w

**Published:** 2022-04-19

**Authors:** Lenny Gallardo-Alvarado, David Cantú-de León, Rebeca Ramirez-Morales, Gabriel Santiago-Concha, Salim Barquet-Muñoz, Rosa Salcedo-Hernandez, Cinthya Reyes, Sandra Perez-Alvarez, Delia Perez-Montiel, Carlos Perez-Plasencia, Elizabeth Trejo-Duran, Juan Pablo Galicia

**Affiliations:** 1 Programa de Maestría Y Doctorado en Ciencias Médicas, Odontológicas Y de La Salud. UNAM. Mexico City, Mexico City, Mexico; 2 Subdirección de Investigación Clínica, Instituto Nacional de Cancerología, Mexico City, Mexico; 3 Dirección de Investigación, Instituto Nacional de Cancerología, Mexico City, Mexico; 4 Departamento de Radioterapia, Instituto Nacional de Cancerología, Mexico City, Mexico; 5 Departamento de Ginecología, Instituto Nacional de Cancerología, Mexico City, Mexico; 6 Departamento de Patología, Instituto Nacional de Cancerología, Mexico City, Mexico; 7 Laboratorio de Genómica, Instituto Nacional de Cancerología, Mexico City, Mexico

**Correction to: BMC Cancer 22, 1-8 (2022)**

**https://doi.org/10.1186/s12885-022-09506-3**

Following publication of the original article [1], an error was identified in the equal contributions statement. The correct statement should read:

Lenny Gallardo-Alvarado and David Cantú-de León contributed equally to the present study

The original article [1] has been corrected.
